# Immunotherapy in Liver Transplant Recipients with Advanced Solid Malignancies: A Three-Patient Case Series

**DOI:** 10.3390/curroncol33100623

**Published:** 2026-10-16

**Authors:** Belinda Jiao, Charles Vincent Rajadurai, Nissim Benizri, Arielle Sabbah, Scott Owen, Khashayar Esfahani, Marc Deschenes, Philip Wong, Xun Zhao, Jamil Asselah

**Affiliations:** 1 Gerald Bronfman Department of Oncology, McGill University, Montreal, QC H4A 3T2, Canada; belinda.jiao@mail.mcgill.ca (B.J.);; 2 Département d’oncologie médicale, Institut Gustave Roussy, 94805 Villejuif, France; 3 Faculty of Medicine and Health Sciences, University of Sherbrooke, Sherbrooke, QC J1H 5H3, Canada; 4 Cedars Cancer Centre, McGill University Health Centre, Montreal, QC H4A 3J1, Canada; 5 Department of Oncology, St. Mary’s Hospital Center, Montreal, QC H3T 0A2, Canada; 6 Division of Gastroenterology & Hepatology, McGill University, Montreal, QC H4A 3J1, Canada

**Keywords:** immunotherapy, liver transplantation, allograft rejection, advanced solid malignancy, case series

## Abstract

The use of immune checkpoint inhibitors (ICIs) after liver transplantation (LT) remains controversial, because immune activation may cause allograft rejection. Evidence in non-hepatocellular carcinoma (HCC) malignancies is limited. We retrospectively reviewed three LT recipients treated with pembrolizumab for advanced non-HCC malignancies at the McGill University Health Centre; treatment began between May 2020 and January 2025, and follow-up was censored on 31 January 2026. A patient with PD-L1-negative non-small cell lung cancer (NSCLC) achieved stable disease for approximately seven months, whereas a patient with PD-L1-high NSCLC achieved a durable partial response with progression-free survival of approximately 36 months. A third patient with cutaneous squamous cell carcinoma achieved a near-complete radiologic and metabolic response after two cycles. Pembrolizumab was held after this response; subsequent liver-test abnormalities raised concern for graft injury, but biopsy showed no histologic rejection and supported treatment resumption. No clinical or biochemical evidence of rejection was observed in Cases 1 and 2. At cutoff, Case 3 was alive and receiving pembrolizumab after seven cycles. These cases support a cancer-specific, individualized risk-benefit assessment in which expected oncologic benefit, alternatives, graft history, immunosuppression, and acceptance of graft-loss risk guide multidisciplinary treatment and close graft surveillance.

## Simple Summary

Immune checkpoint inhibitors can produce durable cancer responses, but they are rarely used after liver transplantation because they may trigger rejection of the transplanted liver. We describe three liver transplant recipients treated with pembrolizumab for advanced lung or skin cancer. The anticancer outcomes ranged from stable disease to a durable partial response and a near-complete clinical and radiologic response. No clinical or biochemical evidence of rejection occurred in two patients, and liver biopsy excluded rejection in the third when liver tests became abnormal. These findings do not establish that treatment is safe, because all three patients were highly selected and two began immunotherapy more than 15 years after transplantation. Treatment decisions should therefore be individualized and made jointly by oncology and transplant teams, with close graft monitoring and prompt investigation of abnormalities.

## 1. Introduction

Liver transplantation provides durable long-term survival for patients with end-stage liver disease and selected malignancies [1]. As survival improves, an increasing number of LT recipients are living long enough to develop de novo malignancies, with the incidence of solid tumors estimated to be two- to five-fold higher than in the general population. Among these, non-hepatocellular carcinoma (non-HCC) solid malignancies represent a growing and clinically significant challenge [2,3,4,5,6].

The management of malignancy in LT recipients is complex because chronic immunosuppression impairs tumor immune surveillance while maintaining allograft tolerance [7,8]. ICIs targeting the PD-1/PD-L1 and CTLA-4 pathways have transformed the treatment of advanced malignancies by restoring antitumor immunity and are now widely used across multiple cancer types [9]. However, their use in LT recipients remains controversial because immune activation may precipitate allograft rejection [10,11].

The potential oncologic benefit is substantial: ICI-based regimens have produced survival gains over chemotherapy alone in the two malignancies represented in this series. Pembrolizumab-based regimens have improved long-term outcomes in advanced non-small cell lung cancer (NSCLC), particularly in tumors with high PD-L1 expression and when combined with chemotherapy [12,13,14,15]. In metastatic or recurrent cutaneous squamous cell carcinoma (cSCC), where historical mortality with distant metastatic disease exceeds 70% [16], PD-1 blockade has produced durable responses, with a 36-month overall survival of approximately 40% in recurrent or metastatic disease in KEYNOTE-629 [17,18,19]. It is this magnitude of oncologic benefit, weighed against the possibility of irreversible graft loss, that underlies the decision to offer ICI therapy to transplant recipients despite the absence of transplant-specific efficacy data.

A systematic review and individual participant data meta-analysis by Saleem et al. reported an overall allograft rejection rate of 36.2% among multiple solid organ transplant recipients treated with ICIs [20]. However, more recently, a prospective multicentre study using a standardized immunosuppression protocol reported a rejection rate of approximately 5% in carefully selected LT recipients [21]. Although encouraging, these findings remain insufficient to support broadly applicable clinical algorithms.

An expert consensus from the Italian Association for the Study of the Liver (AISF) highlights that current evidence is sparse, heterogeneous, and largely retrospective, with no standardized guidance regarding patient selection, timing of therapy, immunosuppressive strategies, or long-term graft and oncologic outcome [22]. This knowledge gap is even more pronounced in LT recipients with non-HCC malignancies.

We present three LT recipients treated with pembrolizumab for advanced non-HCC malignancies at the McGill University Health Centre (MUHC). This series adds detailed longitudinal experience from two NSCLC cases with contrasting PD-L1 biology and one metastatic cSCC, linking cancer-specific outcomes to immunosuppressive management and graft outcomes.

## 2. Case Descriptions and Methods

Given the rarity of ICI use after LT, this series represents a selected clinical sample identified through treating-physician recall within the McGill University Health Centre (MUHC) oncology and Multi-Organ Transplant Program rather than through registry or database screening. Included patients were adult LT recipients with an advanced non-HCC solid malignancy who received at least one ICI dose between May 2020 and January 2025 and had sufficiently complete longitudinal oncology and transplant records through the prespecified cutoff of 31 January 2026. Because ascertainment was neither systematic nor consecutive, the total number of eligible patients and the number or reasons for exclusions could not be determined.

Data were abstracted from electronic medical records, including clinical notes, treatment records, imaging, pathology, laboratory results, and immunosuppressive treatment histories. Best documented tumor response was based on routine clinical imaging and examination without formal RECIST 1.1 adjudication. Progression-free survival (PFS) and overall survival (OS) were measured from the first immune checkpoint inhibitor dose, with patients alive at the cutoff censored on that date. Graft injury was suspected when new liver-test abnormalities or other clinical findings prompted evaluation. Rejection was considered confirmed only when supported histologically by allograft biopsy. In patients without biopsy, no rejection was recorded when there was no clinical or biochemical evidence through the last follow-up, although subclinical rejection could not be excluded. The series is reported in accordance with the CARE (CAse REport) guidelines [23].

### 2.1. Case 1

A woman in her late 70s underwent orthotopic liver transplantation for decompensated cirrhosis secondary to metabolic dysfunction-associated steatohepatitis (MASH) and was maintained on tacrolimus monotherapy (0.5 mg each morning and 1 mg each evening), with stable graft function. Her history included heavy tobacco exposure and two synchronous primary lung malignancies diagnosed approximately eight years after transplantation: a pT2aN0 adenocarcinoma of the right upper lobe treated with lobectomy and a pT1bNx squamous cell carcinoma of the left upper lobe treated several months later with wedge resection. Molecular testing identified no actionable oncogenic alterations. She received no adjuvant therapy after either resection.

Approximately 14 years after transplantation, biopsy-confirmed locoregional recurrence of poorly differentiated NSCLC with adenocarcinoma features was identified in the left upper lobe. PD-L1 expression was 0%. Stereotactic body radiotherapy (50 Gy in five fractions) achieved initial control. Approximately two years later, surveillance imaging demonstrated new osseous metastases, and subsequent Positron Emission Tomography (PET) scan showed multifocal skeletal and hepatic metastases. Biopsy of the liver allograft confirmed metastatic poorly differentiated carcinoma consistent with a lung primary. Next-generation sequencing (NGS) with the AmpliSeq Focus panel identified no actionable genomic alterations.

First-line carboplatin with an area under the curve (AUC) dose of 4, pemetrexed (initially at 80% of the standard dose), and pembrolizumab (2 mg/kg) every three weeks was initiated approximately 16 years after transplantation. After four induction cycles, the patient transitioned to pemetrexed plus pembrolizumab maintenance on the same three-week schedule and achieved stable disease as the best documented response. She received 11 cycles in total, including four induction cycles and seven maintenance cycles, over approximately seven months. Subsequent Computed Tomography (CT) imaging demonstrated progressive hepatic metastases, while allograft function remained preserved and no clinical or biochemical evidence of rejection was observed. With disease progression and declining performance status, she transitioned to best supportive care.

### 2.2. Case 2

A man in his 70s who had undergone orthotopic liver transplantation for primary biliary cirrhosis presented approximately 17 years later with metastatic NSCLC. His immunosuppressive regimen consisted of mycophenolate mofetil (MMF) 500 mg twice daily and cyclosporine, monitored using C2 concentrations obtained two hours after administration. His history included a 40-pack-year smoking exposure and a previously resected cutaneous basal cell carcinoma.

Progressive dyspnea and fatigue prompted imaging, which identified a suspicious lung nodule. Biopsy confirmed pulmonary adenocarcinoma with PD-L1 expression of 80% and a KRAS G12C mutation on NGS (AmpliSeq Focus). Staging CT demonstrated extensive metastatic disease, including mediastinal lymphadenopathy, pericardial infiltration with early tamponade physiology, and osseous metastases.

Given the aggressive disease and high PD-L1 expression, the patient was treated with carboplatin (AUC 5), pemetrexed (500 mg/m^2^), and pembrolizumab (2 mg/kg) every three weeks, following a multidisciplinary discussion.

Following multidisciplinary review by oncology and transplant hepatology, immunosuppression was adjusted concurrently. MMF was initially withheld to mitigate overlapping hematologic toxicity, and cyclosporine was reduced to 50 mg each morning and 25 mg each evening, with continued C2 monitoring. Cyclosporine was subsequently reduced to 25 mg twice daily while continuing the calcineurin-inhibitor. The patient improved clinically after the first cycle, and a best documented partial response was confirmed after four cycles.

Pemetrexed and pembrolizumab were temporarily paused during an episode of renal failure, when the estimated glomerular filtration rate declined from 58 to 32 mL/min/1.73 m^2^. Following renal recovery, pembrolizumab monotherapy was resumed, whereas pemetrexed was permanently discontinued. Cyclosporine was maintained at 25 mg twice daily because of persistent kidney dysfunction, while MMF was reintroduced at 250 mg twice daily to maintain background immunosuppression. The patient completed 25 cycles of pembrolizumab before treatment was stopped because of fluctuating renal function. Approximately six months later, sirolimus 2 mg daily was added by the transplant team as part of ongoing immunosuppressive management. Allograft function remained stable, with no clinical or biochemical evidence of rejection.

Approximately 20 years after transplantation and 1.5 years after his last dose of ICI, the patient developed superior vena cava syndrome, marking progression at approximately 36 months from ICI initiation and a PFS of approximately 36 months. He received palliative radiotherapy (30 Gy in 10 fractions) and resumed pembrolizumab for an additional 10 cycles, bringing cumulative exposure to 35 cycles, with durable clinical benefit and good clinical and biochemical tolerance. Following a subsequent treatment-free interval, worsening dyspnea prompted imaging that demonstrated progression of a right hilar mass with pulmonary artery invasion. The use of a targeted KRAS G12C inhibitor (such as sotorasib) was considered; however, given the patient’s excellent prior response to ICI-based therapy and the prolonged treatment-free interval before disease recurrence, systemic rechallenge with carboplatin, pemetrexed, and pembrolizumab was favored. Following a single cycle of this rechallenge, the patient experienced rapid respiratory deterioration. He was transitioned to comfort-focused care and died shortly thereafter.

### 2.3. Case 3

A man in his late 60s underwent orthotopic liver transplantation for metabolic dysfunction-associated steatohepatitis (MASH) approximately six years before ICI initiation and was maintained on tacrolimus 1.5 mg twice daily. Beginning approximately two years after transplantation, he developed multiple cutaneous squamous cell carcinomas (cSCCs) requiring serial Mohs micrographic surgeries and wide local excisions. Approximately five months before ICI initiation, the disease progressed to advanced unresectable cSCC, for which oral capecitabine was initiated. PET/CT approximately two months before ICI initiation confirmed widespread metastatic progression, with bilateral scalp lesions, bilateral cervical and supraclavicular lymphadenopathy, mediastinal involvement, new bone metastases, and lung opacities suspicious for metastases. Intravenous carboplatin and paclitaxel were commenced at that time. The immunosuppression plan was developed jointly by oncology and transplant hepatology: tacrolimus was transitioned to sirolimus 3 mg daily to reduce net immunosuppression and draw on the potential antineoplastic properties of mTOR inhibition.

Approximately six years after transplant, pembrolizumab was initiated as maintenance therapy at a dose of 4 mg/kg intravenously every six weeks (maximum 400 mg), in accordance with the institutional weight-based dosing protocol. This schedule provides the same nominal dose intensity as 2 mg/kg every three weeks and was selected by the treating oncologist to reduce treatment-visit frequency. Under the same multidisciplinary plan, a prednisone taper was introduced concurrently with the aim of mitigating allograft rejection risk (40 mg on days 1–4, 20 mg on days 5–6, then 10 mg daily ongoing), alongside continued sirolimus.

CT approximately one month after ICI initiation demonstrated resolution of the previously observed pulmonary opacities and right paratracheal adenopathy. PET/CT approximately two months after initiation showed a near-complete metabolic response, with resolution of right-neck cutaneous disease, cervical and intrathoracic nodal disease, pulmonary lesions, and nearly all osseous metastases. Residual findings consisted of patchy right frontoparietal scalp uptake with a few tiny moderately avid cutaneous or subcutaneous foci and mild uptake at the lesser trochanter.

Following the marked oncologic response, pembrolizumab was suspended approximately two months after initiation. CRP subsequently rose from 24.6 mg/L to 450 mg/L over the following month. Liver tests at that time included total bilirubin 7 μmol/L, ALT 52 U/L, AST 49 U/L, alkaline phosphatase 265 U/L, GGT 176 U/L, and LDH 255 U/L, raising concern for immune-mediated hepatotoxicity or allograft rejection. Liver enzymes fluctuated over the following weeks, with ALT peaking at 95 U/L approximately five months after ICI initiation. Liver biopsy approximately six months after ICI initiation showed mild focal lymphocytic infiltration of portal spaces and lobules and mild iron accumulation in Kupffer cells, with no histologic evidence of allograft rejection. Approximately 10 days later, laboratory values had improved (ALT 28 U/L, AST 27 U/L, alkaline phosphatase 128 U/L, GGT 96 U/L, and CRP 15.3 mg/L).

PET/CT six months after ICI initiation demonstrated recurrent disease, including a hypermetabolic right parieto-occipital scalp lesion with outer-table bone erosion, recurrent T7 bone metastasis, a suspicious left upper-lobe pulmonary nodule, and mediastinal and hilar lymphadenopathy. Hypofractionated radiotherapy to the scalp (25 Gy in five fractions) was completed approximately seven months after ICI initiation and was complicated by a wound infection managed with antibiotics. Pembrolizumab was rechallenged as cycle 3 immediately after completion of scalp radiotherapy. Palliative radiotherapy to T7 (8 Gy in one fraction) was administered approximately 11 months after ICI initiation while pembrolizumab continued, and palliative care was formally integrated at that time.

Approximately 12 months after ICI initiation, the patient was hospitalized with confusion, agitation, hyponatremia (sodium 122 mmol/L; adjusted sodium 127 mmol/L), and hyperglycemia (17.5 mmol/L). Incidental pneumomediastinum without evidence of esophageal perforation was managed conservatively. No biochemical evidence of hepatitis or graft dysfunction was identified.

At the prespecified data cutoff, approximately 12 months after ICI initiation, the patient was alive and receiving active pembrolizumab after seven cycles. Allograft function remained preserved and repeat imaging was pending.

In summary, Cases 1 and 2 initiated ICI therapy approximately 16 and 17 years after LT, respectively, whereas Case 3 initiated treatment approximately six years after LT; all three had stable baseline graft function. These characteristics may have influenced treatment selection and limit generalizability, and no causal relationship with preserved graft function can be inferred. No clinical or biochemical evidence of rejection was observed in Cases 1 and 2, although subclinical rejection could not be excluded. Biopsy in Case 3 showed no histologic rejection. No definite immune-related adverse event was documented in Case 1. In Case 2, renal dysfunction was considered multifactorial, with a possible ICI-related contribution; however, a renal biopsy was not performed, and renal function subsequently improved following treatment adjustments. In Case 3, liver-test abnormalities prompted evaluation for immune-mediated hepatotoxicity and rejection, but biopsy showed neither rejection nor clear histologic evidence of immune-related hepatitis.

**Figure 1:**
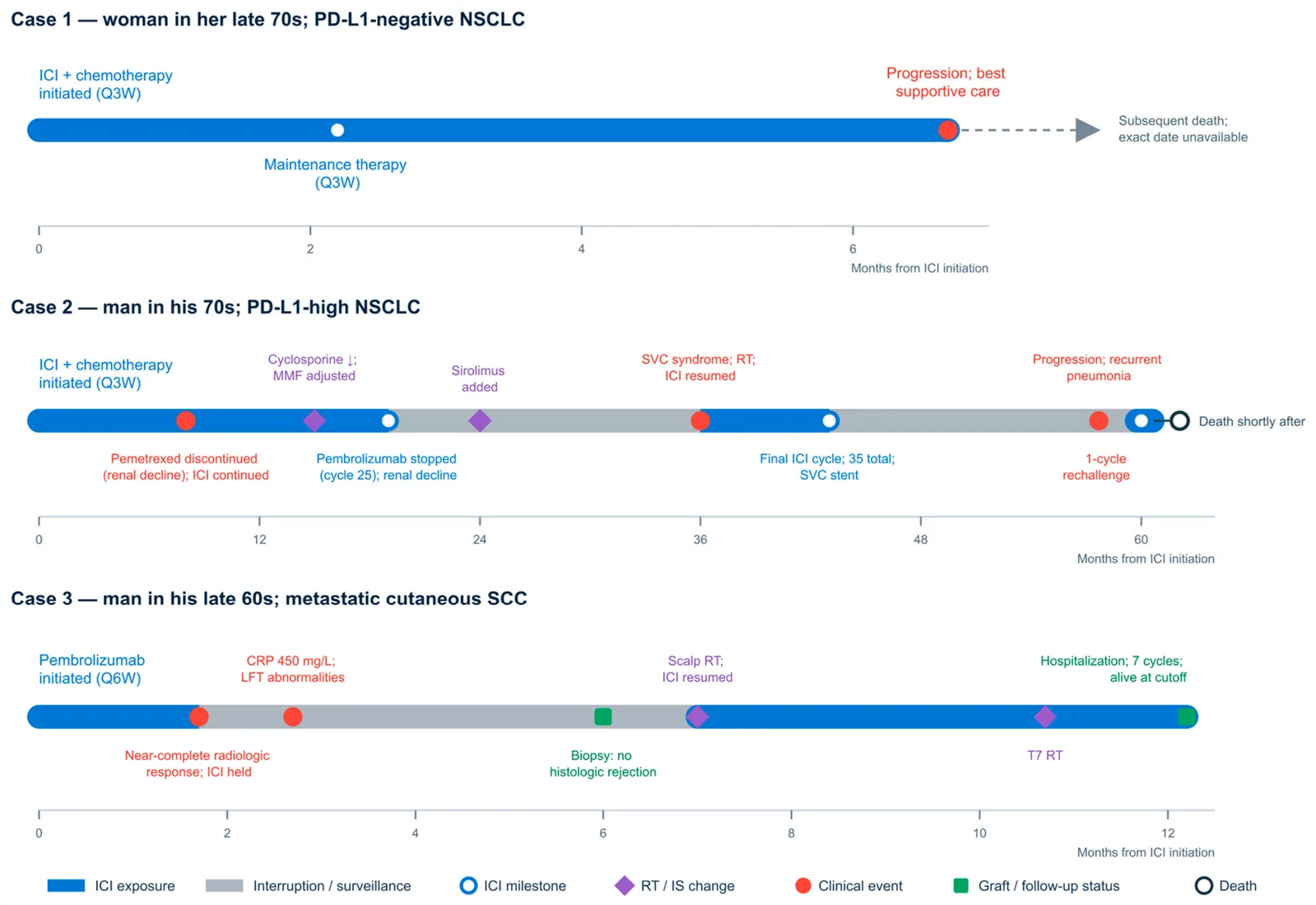
Clinical timelines of the three LT patients treated with ICI. Each panel represents one patient and shows ICI exposure and interruptions, locoregional therapy, immunosuppression changes, key clinical events, graft-related outcomes, and follow-up status. Time is shown in months from ICI initiation (month 0) through the prespecified cutoff of 31 January 2026; scales differ among panels. For Case 2, “35 total” denotes 25 initial pembrolizumab cycles plus 10 cycles after resumption, before the subsequent single-cycle triplet rechallenge. For Case 1, the dashed arrow indicates subsequent death for which the exact date was unavailable and is therefore not plotted to scale. The downward arrow (↓) in Case 2 indicates a reduction in cyclosporine dose. ICI = immune checkpoint inhibitor; RT = radiotherapy; IS = immunosuppression; NSCLC = non-small cell lung cancer; SCC = squamous cell carcinoma; PD-L1 = programmed death-ligand 1; MMF = mycophenolate mofetil; SVC = superior vena cava; CRP = C-reactive protein; LFT = liver function test; Q3W = every 3 weeks; Q6W = every 6 weeks.

ICI was administered alongside differing immunosuppressive strategies, including mTOR inhibitors and corticosteroids, but their effects on antitumor efficacy and allograft outcomes remain uncertain. During ICI therapy, liver biochemistry and immunosuppressant drug concentrations were generally assessed at each treatment cycle or clinical follow-up visit, with monitoring intensified when abnormalities arose or at the discretion of the treating teams. Close biochemical follow-up and early diagnostic evaluation, including allograft biopsy when indicated, were important for distinguishing suspected immune-related injury from rejection. Clinical characteristics, immunosuppression strategies, and oncologic timelines are summarized in Table 1 and Table 2 and Figure 1.

## 3. Discussion

The three cases provide complementary rather than interchangeable observations. All patients received pembrolizumab after LT, but their tumor biology, expected sensitivity to ICI therapy, immunosuppressive management, and oncologic outcomes differed. Case 1 had PD-L1-negative NSCLC and achieved stable disease; Case 2 had PD-L1-high NSCLC and achieved a durable partial response; and Case 3 had metastatic cSCC and achieved a near-complete radiologic response. These contrasting outcomes suggest that the expected cancer-specific benefit remains central to treatment selection. The transplant history modifies the risk-benefit assessment, but it does not remove the importance of tumor type and predictive biomarkers.

No clinical or biochemical evidence of rejection was observed in Cases 1 and 2 although subclinical rejection could not be excluded without a biopsy. In Case 3, a liver biopsy was performed due to clinical suspicion but showed no histologic rejection. This is clinically reassuring but should not be interpreted as evidence that pembrolizumab is safe after LT. Published estimates of rejection vary substantially across selected cohorts, and rejection may cause irreversible graft loss and death [10,11,20,24,25]. The value of this series is therefore not the absence of rejection alone, but the way each case illustrates clinical decision-making encountered when ICI therapy is considered and administered after LT.

### 3.1. Cases 1 and 2: Contrasting NSCLC Outcomes

Case 1 illustrates the narrower therapeutic margin when the expected incremental benefit of ICI therapy is modest. Despite a PD-L1 expression of 0%, combined carboplatin, pemetrexed, and pembrolizumab achieved stable disease for approximately seven months without clinically apparent graft injury. For context, median PFS and OS in the PD-L1-negative subgroup of KEYNOTE-189 were 6.1 and 17.2 months, respectively [13,15]. This outcome shows that disease control is possible in a PD-L1-negative tumor, but the limited duration of benefit is important when weighed against the possibility of catastrophic rejection. In such circumstances, the decision to use an ICI should depend on the expected contribution of immunotherapy, available non-ICI options, disease trajectory, and the patient’s goals rather than on transplant status alone.

Case 2 illustrates an important clinical benefit from treatment. The tumor had PD-L1 expression of 80%, and the patient achieved a radiologic partial response that was maintained across initial therapy, a durable treatment-free interval, and subsequent pembrolizumab re-exposure. Published data in transplant recipients with thoracic malignancies remain limited, but the INNOVATED cohort reported an objective response rate of 45.5% in its lung cancer subgroup, although median OS was only 4.6 months [26]. The approximately 36-month response and PFS in Case 2 therefore represent a clinically meaningful outcome. First-line pembrolizumab-based therapy in the general (non-transplant) NSCLC population confers an approximately two-fold five-year overall survival advantage in high PD-L1 disease and more than doubles median overall survival in PD-L1-unselected non-squamous disease when combined with chemotherapy [14,15]; Cases 1 and 2 span this spectrum, from PD-L1-negative disease with a modest, time-limited benefit to PD-L1-high disease with a multi-year response. These general-population trials excluded solid-organ transplant recipients and therefore are not comparable to the described cases.

The immunosuppressive course in Case 2 also illustrates why graft outcomes cannot be attributed to a single drug. MMF was initially suspended, cyclosporine was dose-reduced, low-dose MMF was later reintroduced, and sirolimus was subsequently added. Allograft function remained stable, but the simultaneous changes, renal dysfunction, and long interval from transplantation make causal interpretation impossible. A French multicentre cohort found that calcineurin inhibitor use at ICI initiation was associated with a lower risk of rejection [27]; notably, Case 2 continued reduced-dose cyclosporine rather than undergoing complete calcineurin inhibitor withdrawal. This concordance is hypothesis-generating, not proof of protection.

### 3.2. Case 3: High Expected Benefit and the Role of Biopsy

Case 3 represents a different risk-benefit context because advanced cSCC is highly responsive to PD-1 blockade in immunocompetent populations [17,18]. For context, in the recurrent or metastatic cohort of KEYNOTE-629, median PFS and OS were 5.7 and 23.8 months, respectively, with an objective response rate of 35.2% [19]. In a pilot study of solid organ transplant recipients with metastatic cSCC, four of the seven evaluable patients responded, and no acute graft rejection was reported, although clinically important toxicities occurred [28]. The near-complete radiologic and metabolic response in Case 3 is consistent with this known disease sensitivity and provides a stronger oncologic justification for balancing graft risk than would be present for a poorly ICI-responsive malignancy.

This case also demonstrates the importance of separating treatment interruption from the later evaluation for graft injury. Pembrolizumab was initially held following the marked oncologic response. Subsequent inflammatory and liver-test abnormalities raised concern for immune-mediated hepatotoxicity or allograft rejection, but biopsy showed only mild focal lymphocytic infiltration without histologic rejection. Abnormal liver tests after ICI exposure are not specific and may reflect rejection, immune-related hepatitis, infection, biliary or vascular complications, medication toxicity, or tumor progression. Biopsy changed management as it prevented the abnormalities from being classified as rejection without histologic support and supported pembrolizumab rechallenge.

The marked inflammatory response in Case 3 should likewise not be treated as a surrogate for rejection. Immune-related adverse events may accompany systemic immune activation, but their ability to predict allograft rejection after transplantation has not been validated [29,30]. Pre-treatment graft PD-L1 expression has been explored as a rejection-risk marker during PD-1 therapy, but the evidence remains preliminary [31]. Donor-derived cell-free DNA has shown potential for detecting general liver-allograft injury and monitoring graft health [32], but it has not been evaluated as a predictor of ICI-associated rejection or as a tool for ICI candidacy. Neither biomarker currently replaces clinical assessment or biopsy when graft injury is suspected.

### 3.3. Current Evidence for ICI Use After Liver Transplantation

Reported rejection risk after ICI therapy in transplant recipients varies substantially across cohorts and cannot yet be reduced to a single actionable estimate. A pooled analysis across solid organ transplant types found rejection in 36.2% of ICI-treated recipients [20], while liver-specific pooled data reported rejection in 28.8% and death from graft loss in 13.4% of cases [24], underscoring the potential severity of this complication. By contrast, a prospective cohort using a standardized clinical practice protocol reported one rejection event among 20 selected LT recipients [21]. This variation likely reflects differences in patient selection, cancer type, immunosuppressive management, and monitoring intensity and argues against applying any single published rate to an individual treatment decision. A global peri-transplant cohort also reported more graft loss or dysfunction with post-transplant than pre-transplant ICI exposure, despite comparable reported rejection rates [33]; differences in population and treatment timing limit direct interpretation.

Time since transplantation is often treated as reassuring, but supporting evidence is limited. Rejection has occurred when ICI therapy was initiated more than four years after LT [34], showing that a long transplant-to-ICI interval does not eliminate risk. Immunosuppressive strategy at ICI initiation may modify risk more directly: a French multicentre cohort found calcineurin inhibitor use at ICI initiation to be associated with a lower risk of rejection [27], and mechanistic and preclinical work has raised the possibility that mTOR inhibition may help preserve antitumor immune activity while limiting alloimmune activation [35,36]. Neither association has been tested prospectively, and both should remain hypothesis-generating rather than serve as a basis for a preferred regimen. Because nearly all published experience involves PD-1/PD-L1 inhibitors in heterogeneous, non-comparable cohorts, the evidence is insufficient to select one ICI class over another on safety grounds [37,38].

Distinguishing rejection from other causes of graft dysfunction during ICI therapy remains a central clinical challenge. When biochemical abnormalities raise concern for graft injury, allograft biopsy remains central to confirming or excluding rejection when clinically feasible.

Together, this evidence supports a cautious and patient-centered approach to ICI use after LT. Because rejection risk is a significant concern and can be fatal, clinical decisions must rely on individualized risk assessments rather than population-level averages, especially since current predictors remain unvalidated.

### 3.4. What the Cases Suggest About Graft Risk and Immunosuppression

The absence of clinically apparent rejection across three different immunosuppressive approaches is notable but not explanatory. Tacrolimus monotherapy was maintained in Case 1; Case 2 underwent sequential modification of cyclosporine, MMF, and sirolimus; and Case 3 was transitioned from tacrolimus to sirolimus with concomitant prednisone. Cases 1 and 2 began ICI therapy approximately 16 and 17 years after LT, respectively, compared with approximately six years for Case 3. The >15-year intervals in Cases 1 and 2 and stable baseline graft function in all three patients may have influenced their selection for ICI therapy but should not be interpreted as protective.

The clinical experience in Cases 2 and 3 cannot establish that sirolimus prevented rejection, and the preserved graft function in Case 1 on tacrolimus monotherapy shows that mTOR-based conversion is not the only context in which ICI treatment may be tolerated. Because all three patients received pembrolizumab, this series also provides no basis for comparing rejection risk among ICI classes.

### 3.5. Clinical Implications, Limitations, and Future Directions

The cases support a cancer-specific and multidisciplinary approach rather than a uniform contraindication to ICI therapy after LT. The strongest rationale exists when the malignancy has a high expected probability of durable response and effective non-ICI alternatives are exhausted or limited. Before treatment, oncology and transplant hepatology should review the graft history, baseline liver function, current immunosuppression, competing treatment options, and the patient’s acceptance of possible irreversible graft loss. During therapy, monitoring should include liver biochemistry and immunosuppressant levels, with early transplant assessment and a low threshold for biopsy when the cause of graft dysfunction is uncertain [21,22].

This series is limited primarily by substantial selection bias, as well as its retrospective descriptive design, small sample size, clinical heterogeneity, absence of a comparator, and use of routine rather than protocol-mandated response and graft assessments. Cases 1 and 2 initiated ICI therapy more than 15 years after LT, and all three patients had stable baseline graft function. The reported patients therefore likely represent a highly selected group considered suitable for treatment. Indeed, recipients earlier after transplantation, with prior rejection, or with less stable graft function may have been less likely to receive ICI therapy and are not represented in this series. Accordingly, the preserved graft outcomes cannot be generalized to all LT recipients considered for ICI therapy, and this series cannot estimate rejection risk, establish a preferred immunosuppressive regimen, or determine whether tumor biomarkers predict benefit in LT recipients as they do in non-transplant populations. Prospective multicentre registries should include both treated and untreated candidates and use standardized definitions of rejection, graft loss, tumor response, and survival. Cancer-specific studies are needed to identify the settings in which expected oncologic benefit may justify graft risk.

## 4. Conclusions

Liver transplantation should not be treated as an absolute contraindication to ICI therapy, but use should remain exceptional, individualized, and multidisciplinary. In this series, pembrolizumab produced outcomes ranging from disease stabilization to durable partial and near-complete responses, without clinical or biochemical evidence of rejection in Cases 1 and 2 or histologic rejection in Case 3; however, subclinical rejection cannot be excluded when biopsy is not performed. These cases illustrate individualized risk–benefit decision-making but cannot establish the safety of ICI therapy, define a safe immunosuppressive regimen, or identify patients at acceptably low risk of rejection. Treatment decisions should be driven by the expected cancer-specific benefit relative to alternatives, while integrating graft history, immunosuppressive strategy, patient acceptance of graft-loss risk, close biochemical surveillance, and prompt biopsy of unexplained graft dysfunction. Until validated predictors of rejection are available, this risk-adapted framework is more defensible than either routine exclusion or broad adoption.

## Figures and Tables

**Table 1:** Patient Characteristics, Oncologic Treatment, and Immunosuppression Strategies.

Characteristic	Case 1	Case 2	Case 3
Patient and transplant background			
Age/Sex	Late 70s/F	70s/M	Late 60s/M
Indication for LT	MASH cirrhosis	Primary biliary cirrhosis	MASH
Baseline immunosuppression	Tacrolimus 0.5 mg AM/1 mg PM	Cyclosporine + MMF 500 mg BID	Tacrolimus 1.5 mg BID
Prior graft rejection	None documented	None documented	None documented
Baseline graft status	Stable liver biochemistry	Stable liver biochemistry	Stable liver biochemistry
Relevant pre-ICI drug level	Tacrolimus trough 3.4 ng/mL	Cyclosporine concentration 649 ng/mL around ICI initiation; monitored using C2 measurements	Tacrolimus trough 7.5 ng/mL before conversion
ECOG performance status at ICI initiation	1	1	1
Oncologic diagnosis			
Cancer type	Recurrent metastatic NSCLC (adenocarcinoma)	Metastatic NSCLC (adenocarcinoma)	Metastatic cutaneous SCC (cSCC)
Key molecular features	PD-L1 0%; no actionable alterations on NGS	PD-L1 80%; KRAS G12C+; ALK/ROS1 negative	Not reported
Metastatic sites at ICI initiation	Bone, liver allograft	Mediastinal LN, pericardium (early tamponade), bone	Scalp (bilateral), bilateral cervical and supraclavicular LN, mediastinal LN, bone (multiple), lung opacities (metastasis vs. inflammatory)—PET ~2 months before ICI start
ICI therapy			
ICI agent	Pembrolizumab	Pembrolizumab	Pembrolizumab
Timing of ICI initiation after LT	~16 years	~17 years	~6 years
ICI exposure	11 cycles (4 induction + 7 maintenance)	35 cycles before later triplet rechallenge (25 initial + 10 after resumption); 1 additional pembrolizumab-containing cycle (36 lifetime cycles)	7 cycles total by 31 January 2026; cycles 1–2 before interruption; cycle 3 resumed ~7 months after ICI initiation
ICI schedule	Q3W throughout	Q3W	Q6W
ICI interruption/rechallenge	None	Stopped after 25 cycles for renal dysfunction; resumed after SVC syndrome for 10 cycles (35 cumulative); later 1 cycle triplet rechallenge at progression	Held ~2–7 months after ICI initiation; resumed after scalp RT; 7 cycles total at cutoff
Immunosuppression management			
IS changes with ICI	No changes; tacrolimus monotherapy maintained throughout	MMF initially withheld; cyclosporine reduced to 50 mg AM/25 mg PM and later to 25 mg BID; low-dose MMF subsequently reintroduced; sirolimus later added	Tacrolimus switched to sirolimus 3 mg daily before ICI; prednisone at ICI start (40 mg days 1–4 → 20 mg days 5–6 → 10 mg daily ongoing)
Available drug levels during follow-up	Tacrolimus trough 5.2 ng/mL at progression	Cyclosporine C2 concentrations 158.7–804 ng/mL (649 ng/mL around ICI initiation); subsequent sirolimus troughs 3.2–11.8 ng/mL	Tacrolimus below 2 ng/mL following conversion; no verified sirolimus level available
mTOR inhibitor use	No	Yes—sirolimus added	Yes—sirolimus as primary IS
Prophylactic corticosteroids	No	No	Yes—prednisone 10 mg/day ongoing
Monitoring strategy	Liver biochemistry and tacrolimus levels at each treatment cycle or follow-up visit; more frequently if clinically indicated.	Liver biochemistry and immunosuppressant levels at each treatment cycle or follow-up visit; more frequently if clinically indicated.	Liver biochemistry and immunosuppressant levels at each treatment cycle or follow-up visit; more frequently if clinically indicated; biopsy performed for subsequent abnormalities.
Locoregional therapy during ICI			
RT delivered	No RT concurrent with ICI (prior SBRT 50 Gy/5 Fx to LUL)	Palliative RT 30 Gy/10 Fx to SVC region, completed before resumption of pembrolizumab	Scalp RT 25 Gy/5 Fx before cycle 3; palliative RT 8 Gy/1 Fx to T7 during pembrolizumab
RT timing relative to ICI	—	Sequential—completed before resuming pembrolizumab	Sequential—scalp RT immediately before ICI rechallenge; T7 RT during pembrolizumab
RT complications	—	None reported	Scalp wound infection after RT ~7 months after ICI start; treated with antibiotics

Abbreviations: ALK, anaplastic lymphoma kinase; AM, morning; BID, twice daily; C2, 2 h post-dose concentration; cSCC, cutaneous squamous cell carcinoma; ECOG, Eastern Cooperative Oncology Group; Fx, fractions; Gy, gray; ICI, immune checkpoint inhibitor; IS, immunosuppression; KRAS, Kirsten rat sarcoma viral oncogene homolog; LN, lymph node; LT, liver transplantation; LUL, left upper lobe; MASH, metabolic dysfunction-associated steatohepatitis; MMF, mycophenolate mofetil; mTOR, mechanistic target of rapamycin; NGS, next-generation sequencing; NSCLC, non-small cell lung cancer; PD-L1, programmed death-ligand 1; PET, positron emission tomography; PM, evening; Q3W, every 3 weeks; Q6W, every 6 weeks; ROS1, ROS proto-oncogene 1 receptor tyrosine kinase; RT, radiotherapy; SBRT, stereotactic body radiotherapy; SCC, squamous cell carcinoma; SVC, superior vena cava.

**Table 2:** Oncologic and Graft Outcomes.

Characteristic	Case 1	Case 2	Case 3
Oncologic outcomes			
Best documented clinical/radiologic response	Stable disease (~7 months)	Durable partial response (~36 months)	Best documented near-complete metabolic response after 2 cycles
PFS from ICI start	~7 months	~36 months	~6.5 months from first ICI exposure to recurrence; ICI held ~2–7 months after ICI start
OS from ICI start	Minimum observed survival ~9 months; exact OS not estimable	~60 months	Alive and censored at ~12 months
Disease course	Progressive liver metastases; best supportive care; subsequent death, exact date unavailable	Durable PR; progression at ~36 months; 10-cycle pembrolizumab resumption; later 1-cycle triplet rechallenge	Near-CR → interruption → recurrence → RT and ICI rechallenge; 7 cycles by cutoff
Status at cutoff	Deceased after disease progression; exact date unavailable	Deceased—respiratory failure	Alive
Graft outcomes			
Allograft rejection	No clinical or biochemical evidence	No clinical or biochemical evidence	No histologic rejection on biopsy
Hepatic events	None; graft function preserved	None; graft function preserved	CRP rose from 24.6 to 450 mg/L; liver-test abnormalities followed (ALT peak 95 U/L); values improved after biopsy showed no rejection; no hepatitis or graft dysfunction at cutoff
Liver biopsy performed	No	No	Yes—~6 months after ICI initiation; mild focal lymphocytic portal and lobular infiltration with mild iron in Kupffer cells; no histologic rejection
Notable complications			
Other events	Renal impairment (pemetrexed dose reduction)	Renal failure/AKI (temporary pembrolizumab pause; pemetrexed discontinued); SVC syndrome (RT); recurrent pneumonia	Scalp wound infection after RT; hyponatremia, hyperglycemia, and pneumomediastinum (~12 months after ICI initiation); palliative care involvement

Abbreviations: AKI, acute kidney injury; ALT, alanine aminotransferase; CR, complete response; CRP, C-reactive protein; ICI, immune checkpoint inhibitor; OS, overall survival; PFS, progression-free survival; PR, partial response; RT, radiotherapy; SVC, superior vena cava.

## Data Availability

The data presented in this study are not publicly available due to patient privacy and confidentiality restrictions. De-identified data may be made available by the corresponding author upon reasonable request, subject to approval by the McGill University Health Centre Research Ethics Board.
